# Identification and characterization of *MAGO* and *Y14* genes in *Hevea brasiliensis*

**DOI:** 10.1590/1678-4685-GMB-2014-0387

**Published:** 2016

**Authors:** Zi-Ping Yang, Hui-Liang Li, Dong Guo, Shi-Qing Peng

**Affiliations:** 1 Key Laboratory of Biology and Genetic Resources of Tropical Crops, Ministry of Agriculture, Institute of Tropical Bioscience and Biotechnology, Chinese Academy of Tropical Agricultural Sciences, Haikou, China; 2 College of Agriculture, Hainan University, Haikou, China

**Keywords:** interaction, Hevea brasiliensis, Mago nashi, Y14 proteins

## Abstract

Mago nashi (MAGO) and Y14 proteins are highly conserved among eukaryotes. In this study, we identified two MAGO (designated as HbMAGO1 and*HbMAGO2*) and two Y14 (designated as *HbY14a*and *HbY14b*) genes in the rubber tree (*Hevea brasiliensis*) genome annotation. Multiple amino acid sequence alignments predicted that HbMAGO and HbY14 proteins are structurally similar to homologous proteins from other species. Tissue-specific expression profiles showed that HbMAGO and *HbY14* genes were expressed in at least one of the tissues (bark, flower, latex, leaf and root) examined. HbMAGOs and HbY14s were predominately located in the nucleus and were found to interact in yeast two-hybrid analysis (YTH) and bimolecular fluorescence complementation (BiFC) assays. HbMAGOs and HbY14s showed the highest transcription in latex and were regulated by ethylene and jasmonate. Interaction between HbMAGO2 and gp91^phox^ (a large subunit of nicotinamide adenine dinucleotide phosphate) was identified using YTH and BiFC assays. These findings suggested that HbMAGO may be involved in the aggregation of rubber particles in *H. brasiliensis*.

## Introduction

The exon junction complex (EJC) regulates post-transcriptional events that include mRNA intracellular export, cytoplasmic localization, non-sense mediated mRNA decay (NMD) and translation enhancement in metazoans (Park and Muench, 2007; Lee *et al.*, 2009; Ashton-Beaucage*et al*., 2010; Roignant and Treisman, 2010; Boothby and Wolniak, 2011; Mufarrege *et al.*, 2011). The EJC is a multiprotein complex assembled onto mRNAs as a consequence of splicing and is thought to provide a molecular link between splicing and post-splicing mRNA metabolism 20-24 bases upstream of mRNA exon-exon junctions (Le *et al.*, 2000, 2003; Tange *et al.*, 2004; Gehring *et al.*, 2009). Several of the EJC components have been identified and classified into two groups, namely, EJC "core" and "peripheral" factors. Four proteins form the EJC core: MAGO (short form of MAGO NASHI and known as MAGOH in humans), Y14 (also known as Tsunagi or RBM8A), eIF4A/eIF4A3 and BTZ (short form of Barentsz, also known as MLN51), of which MAGO and Y14 constitute a stable heterodimeric complex *in vitro* and*in vivo* (Tange *et al.*, 2004, 2005;Le and Seraphin, 2008). Additional components of the EJC include the splicing factors SRm160, Pinin and RnpS1, the NMD factors Upf1, Upf2 and Upf3, the translation factors Skar and Pym, and the export factors UAP56 and REF/Aly (Roignant and Treisman, 2010). These proteins are considered to be peripheral EJC components that are transiently associated with the EJC core in the nucleus or cytoplasm, where they are involved in splicing, export, translation and NMD (Lejeune and Maquat, 2005; Tange*et al.*, 2005).

The MAGO (meaning "no grandchildren" in Japanese) gene was first identified as a strict maternal effect gene in *Drosophila* (Boswell *et al.*, 1991; Newmark and Boswell, 1994; Micklem *et al.*, 1997; Newmark *et al.*, 1997). MAGO can interact with an RNA binding protein, Y14, to regulate cell growth and is expressed throughout the organism (Zhao *et al.*, 1998,^2000^; Le*et al.*, 2001; Mohr*et al.*, 2001; Chen*et al.*, 2007). Based on the crystal structures of the *Drosophila* and human MAGO-Y14 complex, MAGO and Y14 proteins are core components of the EJC and can form a stable heterodimer that strongly associates with spliced mRNA (Lau *et al.*, 2003; Shi and Xu, 2003). Both MAGO and Y14 are evolutionarily highly conserved proteins (Hachet and Ephrussi, 2001; Mohr *et al.*, 2001) and slow co-evolution of the two protein families is required for the maintenance of their obligate heterodimerization mode (Gong *et al.*, 2014). Comparative sequence analysis reveals that MAGO is devoid of known structural motifs while Y14 contains a central RNA-binding domain (Kataoka *et al.*, 2000;Kim *et al.*, 2001; Shi and Xu, 2003). The crystal structure of the heterodimeric complexes, formed in the absence of RNA, shows that the RNA-binding motif is used for Y14/MAGO protein-protein interaction and is not readily available for binding RNA (Fribourg *et al.*, 2003; Lau *et al.*, 2003; Shi and Xu, 2003).

In plants, MAGO proteins have been studied to varying degrees (Chen *et al.*, 2007; Gong and He, 2014). The orthologs of MAGO genes are linked to male fertility. For example, PFMAGO proteins interact with MADS-domain protein MPF2 and are responsible for male fertility and calyx development in*Physalis* (He *et al.*, 2007). In *Arabidopsis,* AtMAGO is required for pollen grain development (Johnson*et al.*, 2004) and embryo development, as well as meristem cell death events (Park *et al.*, 2009). MAGO is essential for spermatogenesis as shown by RNAi knockdown of *MvMAGO* in *Marsilea* (van der Weele *et al.*, 2007;Boothby and Wolniak, 2011). MAGO genes are also involved in the development of other organs, such as leaves, stems, roots and seeds. The overexpression of *TcMAGO* in transgenic tobacco plants results in longer roots and a more complex root system (Chen *et al.*, 2007). In*Arabidopsis*, RNAi-*AtMAGO* plants produce a greater number of leaves, short and fasciated stems, short lateral roots and non-viable seeds (Park *et al.*, 2009). *OsMAGO* knockdown generated short rice plants with abnormal flowers (Gong and He, 2014). The function of MAGO as a component of the EJC is well-understood, and some functions of MAGO have been reported for other plants; however, the existence of a duplicated MAGO gene in rubber trees is not widely known.

The rubber tree (*Hevea brasiliensis*) is the main renewable world-wide source of commercial natural rubber (NR), a biopolymer of high economic interest composed mainly of cis-1,4-polyisoprene. Latex is produced by laticifers after tapping and is a white cytoplasmic colloidal suspension containing mainly rubber particles, but also non-rubber particles, organelles, proteins and serum. In this study, the MAGO and Y14 gene families in rubber trees were characterized. To investigate their evolutionary relationships and functions, we undertook structural and phylogenetic analyses and examined the subcellular locations of these proteins. The expression profiles of MAGOs and Y14s in various organs and the response to ethylene (ET) and jasmonate (JA) were also studied. Furthermore, the interaction partners of HbMAGO were identified in latex and the HbMAGO2 interaction with gp91^phox^ (a large subunit of nicotinamide adenine dinucleotide phosphate) was demonstrated using yeast two-hybrid analysis (YTH) and bimolecular fluorescence complementation (BiFC) assays. Our results indicate that HbMAGO may be involved in the aggregation of natural rubber in rubber trees.

## Materials and Methods

### Plant materials

*Hevea brasiliensis* cultivar RY7-33-97 was planted on the experimental farm of the Chinese Academy of Tropical Agriculture Sciences, Hainan, China. The shoots were treated with 0.5% ethylene (ET) or 0.1% jasmonic acid (JA), as described by Hao and Wu (2000). Latex samples were collected 1, 3, 6, 9, 24 and 48 h after treatment from 12 shoots for each interval and were immediately stored at −80 °C for RNA extraction. Three independent biological replicates were done for each treatment. For latex RNA extraction, the latex was dropped directly into liquid nitrogen in an ice kettle. Rubber tree flowers, leaves and bark were washed with double-distilled water to remove latex and then immediately frozen in liquid nitrogen.

### Database search and sequence conservation analysis of rubber tree MAGO and Y14 genes

A local whole genome shotgun database for rubber tree was established by Yang *et al.* (2014). The full-length cDNA of HbMAGO2 was obtained from a yeast two-hybrid cDNA library of*H. brasiliensis* laticifers using HbWRKY as the bait in the yeast two-hybrid assay (data unpublished). HbY14a was obtained from the yeast two-hybrid cDNA library of *H. brasiliensis* laticifers using HbMAGO2asthe bait. HbMAGO2 and HbY14a were used as queries in Blast searches of the rubber tree whole genome shotgun database. The shotgun sequences that produced the highest significant alignments judged by the scores and E-values were used for HMM-based gene structure prediction (http://linux1.softberry.com/berry.phtml) based on the*Arabidopsis* and rubber tree databases. The genomic DNA and cDNA sequences of four genes were amplified using appropriate primers (developed based on gene structure prediction) and sequenced.

### Phylogenetic analysis and genomic structure

Analysis and comparison of the HbMAGO and HbY14 sequences were done using BLAST at the NCBI database. Clustal X (http://www.ebi.ac.uk/clustalw) was used for multiple alignments of the nucleic acid and amino acid sequences of HbMAGOs and HbY14s. A phylogenetic tree was constructed with MEGA5.2 (http://www.megasoftware.net/) using the maximum likelihood (ML) method with a bootstrap parameter of 1000 replicates. The sequence information for a number of homologs from other species was retrieved from the NCBI database and is shown in the explanatory text of Figure 2. Genomic structures of the HbMAGOs and HbY14s were analyzed by comparing the cDNA sequences using GSDS software (http://gsds.cbi.pku.edu.cn/) and the corresponding genomic DNA sequences that were extracted from the local whole genome shotgun database.

### Transient protein expression

To determine the subcellular location of the proteins, full-length cDNAs were cloned (see Table 1 for primers) and inserted into the vector pCAMBIAC1302 driven by the 35S promoter. The resulting pCAMBIA1302 constructs were first transformed into the GV3101 strain of*Agrobacterium tumefaciens* by electroporation (Gene Pulser Xcell, Bio-Rad, USA) and then introduced into onion epidermal cells by agroinfiltration using a water circulating vacuum pump (model SHZ-III B, Shanghai, China) (Yang *et al,*2000, 2014). The empty GFP vector was infiltrated as a control. For the BiFC assay, the open reading frames of HbMAGO2 and gp91^phox^ (without their stop codons) were subcloned into pSPYNE-35S (split YFP N-terminal fragment expression) or pSPYCE-35S (split YFP C-terminal fragment expression) vectors driven by the 35S promoter (seeTable 1 for primers). The resulting pSPYNE and pSPYCE plasmids were transformed into the GV3101 strain and the YNE- and YCE-fused proteins were then co-expressed in onion epidermal cells by agroinfiltration (Walter *et al,*2004; Yang *et al.,*2014). Co-expressions of target genes and YFPN or YFPC were used as negative controls. The cells were dipped in 30% sucrose solution for 30 min to induce plasmolysis and the green fluorescent protein (GFP) or yellow fluorescent protein (YFP) signal was then examined with a confocal laser scanning microscope (Zeiss LSM510, Germany).

**Table 1:** The primer sequences used in this study.

		Forward primer (5'- 3')	Reverse primer (5'- 3')
For qRT-PCR assay	*HbMAGOl*	GCTGTCCATTCGATTTGTATCC	CAGTTCTCGAAAGGCAGTAA
	*HMAGO2*	GGAACTGTTTGAGCTGTGTAATG	TCATAGCATTAGCATTTCCGTTTC
	*HbY14a*	ATCCCTCACTTCTCTCCTATCC	TTGTCTCACCGCTAAATGGG
	*HbY14b*	GTGTTGTTAGAGGGTGGCTATAA	GCCACAGAATTGGAAGCTAGA
	*HbActin*	CAGTGTCTGGATAGGAGGATCTA	AAATGGACCGGACTCATCATAC
	*HbMAGO1-BK/AD*	AGAGAATTCATGATGATGTTTGATGAGATG	TGTGGATCCGAACCAGCCTGAAACATCCTG
	*HbMAGO2-BK/AD*	AGAGAATTCATGTTGAAGATGATGGAAGTT	TGTGGATCCGAACCAGCCTGAAACATCCTG
	*HbY14a-BK/AD*	ACAGAATTCATGCGGTTTCCTCTAGAGAAAGG	TGTGGATCCGAACCAGCCTGAAACATCCTG
	*HbY14b-BK/AD*	ACAGAATTCATGTACCCTGATTCCTGTCCACCA	TGTGGATCCGAACCAGCCTGAAACATCCTG
For vector constructs	*gp91-AD*	ACAGAATTCATGGTTCAATGTCCTTACAGCCA	TGTGGATCCGAACCAGCCTGAAACATCCTG
	*MAGO1-1302*	AGAGAATTCATGATGATGTTTGATGAGATG	TGTGGATCCATATTTTAAGATACCATCTTG
	*MAGO2-1302*	ACAGAATTCATGGTCCCAGGAGCAGGCTAG	TGTGGATCCGAACCAGCCTGAAACATCCTG
	*Y14a-1302*	AGAGAATTCATGATGATGTTTGATGAGATG	TGTGGATCCTGCCGCTGCTTAGCAATGTCG
	*Y14b-1302*	ACACCATGGATGGCTAACGCGGATGCGGAA	TGTACTAGTGTATCTCCTCCTAGGACTCCT
	*HbMAGO2-YCE/YNE*	ACATCTAGAATGGCAATGGCAGCGGAAGAT	TGTACTAGTAATAGGCTTGATCTTGAAATG
	*gp91-YNE/YCE*	ACAGTCGACATGAAGGGCTTACCGAAACAT	TGTCCCGGGGCTAAAGTTGTTGCTAATTGA

### Expression analysis

Total RNA was extracted as described by Tang*et al.* (2007). First-strand cDNA was synthesized using a RevertAid™ first-strand cDNA synthesis kit (Fermentas, Lithuania). qRT-PCR was done using the primers indicated in Table 1, with the rubber tree actin gene (GenBank HQ260674.1) being used as a reference gene. The qPCR fragments were amplified and sequenced to determine primer specificity. qRT-PCR was done using the fluorescent dye SYBR-Green (Takara, China) and the melting curves of the amplification products were assessed using a Stratagene Mx3005P real-time thermal cycler (Agilent, America). The qRT-PCR conditions were as follows: 30 s at 95 °C for denaturation followed by 40 cycles of 5 s at 94 °C, 20 s at 56 °C and 20 s at 72 °C for amplification. An average of three independent biological replicates was run for each time interval. The experimental results were reported as the mean **+** SEM of three repli-cates. The data were analyzed with one-way ANOVA and the level of significance was set at p < 0.01 or p < 0.05 (Fisher's protected least significant difference). All data analyses were done using Statistical Product and Service Solutions software (SPSS, version 16.0).

### Yeast two-hybrid assays

For yeast mating, the open reading frame of HbMAGO2 was inserted into pGBKT7 vectors to create a bait plasmid. The bait plasmid (*pGBKT7-HbMAGO2*) was transformed into the yeast strain AH109 by the lithium acetate method, according to the Yeastmaker yeast transformation system 2 user manual (Clontech); the yeast cells were subsequently grown on SD/-Trp medium for 3 days. The bait strain was co-incubated with the library strain to promote fusion in 2YPDA liquid medium containing kanamycin (50 μg/mL) and the intermixture was shaken slowly at 30 °C. If zygotes were present, the mated culture was centrifuged and resuspended in 0.5YPDA/Kan liquid medium followed by plating on DDO/X/A (double dropout medium: SD/-Trp/-Leu with X-α-Gal and aureobasidin A) medium and incubation at 30 °C for 3-5 days. Plasmids were rescued from yeast, transformed into *E. coli* and the DNA then purified. The interaction was confirmed by co-transforming the library plasmid and bait into the yeast strain AH109. The cDNA inserts were subsequently sequenced to confirm their identity.

To confirm one-to-one interaction, the open reading frame of HbMAGOs, HbY14s and gp91^phox^ were inserted into pGBKT7 or pGADT7 vectors to create bait or prey (primers listed in Table 1). The indicated combination of the bait and prey plasmids was co-transformed into yeast strain AH109 and the yeast cells were plated onto QDO/X/A medium (quadruple dropout medium: SD/-Trp/-Leu/-Ade/-His with X-α-Gal and aureobasidin A). β-Galactosidase activity was measured with o-nitro-phenyl-β-D-galactopyranoside (ONPG) as the substrate according to the Yeast Protocols Handbook (no. PT3024-1, Clontech). The changes in absorbance were monitored at OD_420nm_. The experimental results were reported as the means ± SEM of three repli-cates. The data were analyzed with one-way ANOVA and the level of significance was set at p < 0.01 or p < 0.05 (Fisher's protected least significant difference). All data analyses were done using Statistical Product and Service Solutions software (SPSS, version 16.0).

## Results

### Identification and sequence conservation of rubber tree MAGO and Y14 genes

**Figure 1:**
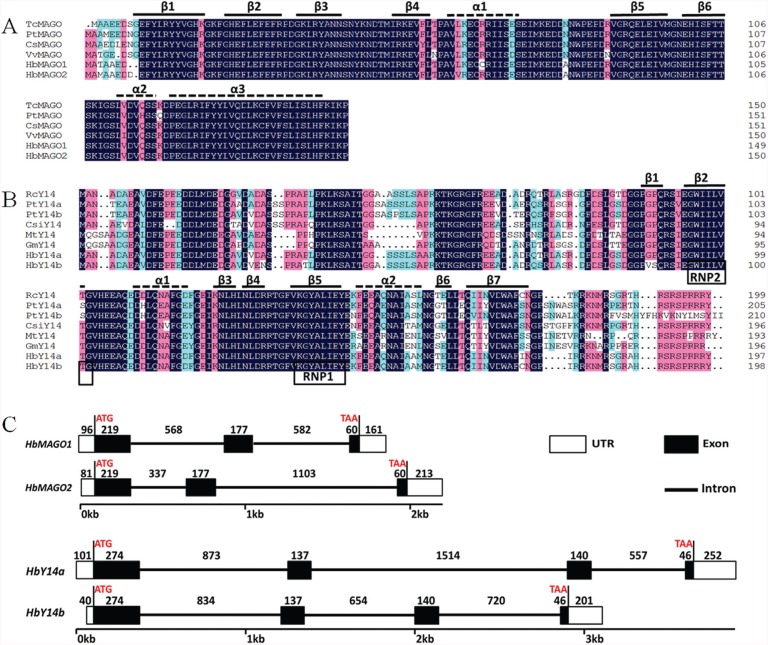
Sequence alignment of the deduced HbMAGOs (A) and HbY14s (B), and intron-exon organization and exon length (C). Amino acid residues that are identical in the sequences are darkly shaded and well-conserved residues are shaded in pink. The α-helices and β-strands of the HbMAGOs and HbY14s are shown as dashed lines and solid lines, respectively. The exons are shown as boxes (open reading frame in black, untranslated region (UTR) in white) and the introns are represented by lines.

Two MAGO genes (designated as HbMAGO1 and *HbMAGO2)* and two Y14 genes (designated as *HbY14a* and *HbY14b)* were identified in rubber tree. The HbMAGO1/2 proteins had calculated molecular masses of 17.476 and 17.688 kDa and isoelectric points (pI) of 5.68 and 5.69 (Table 2). Alignment analysis indicated significant similarities among homologous proteins. The most striking feature of MAGO proteins was their highly conserved primary amino acid sequences (Figure 1A). The HbMAGO1 protein shared 94%, 93%, 92% and 92% amino acid identity with the homologs TcMAGO, PtMAGO, CsMAGO and VvMAGO, respectively; the HbMAGO2 protein shared 94%, 91%, 92% and 91% amino acid identity with the homologs of TcMAGO, PtMAGO, CsMAGO and VvMAGO, respectively. The secondary structure prediction of the MAGO protein revealed that it consisted of six β-strands (β1-β6) and three α-helices (α1-α3) in a β1-β2-β3-β4-α1-β5-β6-α2-α3 arrangement (Figure 1A). The HbY14a/b proteins had calculated molecular masses of 21.737 kDa and 21.903 kDa and isoelectric points (pI) of 4.79 and 4.79 (Table 2).

**Table 2:** Characterization of identified HbMAGO and HbY14 families.

	GenBank	Gene	Exon	Exon length (bp)					ORF	Protein		
Gene name	(AJJZ010000000)	(bp)	number	E1	E2	E3	E4	3'UTR	(bp)	aa	MW	pI
*HbMAGO1*	AJJZ010970869.1 AJJZ011020394.1	1863	3	219	177	60		161	456	151	17,476	5.68
*HbMAGO2*	AJJZ010203456.1 AJJZ010251509.1	2190	3	219	177	60		213	456	151	17,688	5.69
*HbY14a*	AJJZ010172128.1 AJJZ010296629.1	3894	4	274	137	140	46	252	597	198	21,737	4.79
*HbY14b*	AJJZ010209074.1 AJJZ010209073.1	3046	4	274	137	140	46	201	597	198	21,903	4.79

aa – amino acids, ORF – open reading frame.

The HbY14a protein shared 92%, 80%, 72%, 77%, 68% and 72% amino acid identity with the homologs of RcMAGO, PtaMAGO, PtbMAGO, CsiMAGO, MtMAGO and GmMAGO, while the HbY14b protein shared 91%, 80%, 71%, 75%, 67% and 69% amino acid identity with the homologs of RcMAGO, PtaMAGO, PtbMAGO, CsiMAGO, MtMAGO and GmMAGO. The Y14s protein possessed seven β-strands (β1-β7) and two α-helices (α1-α2) in a β1-β2-α1-β3-β4-β5-α2-β6-β7 arrangement (Figure 1B). Alignment of the Y14 proteins revealed a well-conserved RNA-binding domain (RBD) in the middle of the protein (Figure 1B). An exon-intron structure analysis revealed that HbMAGOs consisted of three introns and two exons, while the HbY14s had three introns and four exons, respectively (Figure 1C). Although, the differences in the cDNA sequences between two MAGOs or two Y14s were small, the differences in genomic DNA sequences were enormous.

### Phylogenetic analysis of the rubber tree HbMAGO and HbY14 families

**Figure 2:**
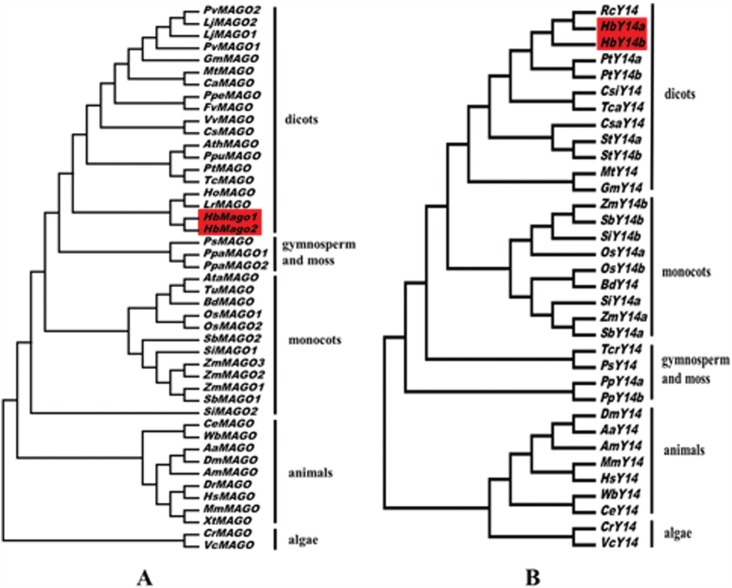
Phylogenetic trees of HbMAGOs (A) and HbY14s (B). The trees were calculated based on the HbMAGO and HbY14 protein sequences and other plant MAGOs and Y14s. The GenBank accession numbers of selected homologs used to produce the phylogenetic trees are: CeMAGO (NP_493025.1), AaMAGO (XP_001660832.1), AmMAGO (XP_001120074.1), DmMAGO (NP_476636.1), WbMAGO (EJW84410.1), HsMAGO (NP_002361.1), MmMAGO (NP_079840.2), DrMAGO (NP_001017700.1), XtMAGO (XP_002931471.1), CrMAGO (XP_001694745.1), VcMAGO (XP_002954749.1), OsMAGO1 (EEC82788.1), OsMAGO2 (NP_001066589.1), ZmMAGO1 (NP_001145913.1), ZmMAGO2 (NP_001146966.1), ZmMAGO3 (ACG28070.1), SiMAGO1 (XP_004972442.1), SiMAGO2 (XP_004957130.1), BdMAGO (XP_003573269.1), AtaMAGO (EMT32727.1), TuMAGO (EMS54552.1), SbMAGO1 (XP_002443724.1), SbMAGO2 (XP_002462539.1), AthMAGO (NP_171716.1), TcMAGO (XP_007052335.1), PtMAGO (XP_006375160.1), LrMAGO (ACT33369.1), PpeMAGO (XP_007220423.1), CsMAGO (XP_004133764.1), PpuMAGO (ABQ11262.1), VvMAGO (XP_002281294.1), CaMAGO (XP_004501210.1), MtMAGO (ACJ86076.1), GmMAGO (NP_001236090.1), LjMAGO1 (AFK33465.1), LjMAGO2 (AFK48815.1), PvMAGO1 (XP_007147870.1), PvMAGO2 (XP_007134432.1), HoMAGO (AAS20975.1), FvMAGO (XP_004306981.1), PsMAGO (ABK22137.1), PpaMAGO1 (XP_001770408.1), PpatMAGO2 (XP_001763801.1), CeY14 (NP_497891.1), AaY14 (XP_001652167.1), AmY14 (XP_395245.2), DmY14 (NP_610454.2), WbY14 (EJW88540.1), HsY14 (NP_005096.1), MmY14 (NP_001095877.1), CrY14 (XP_002953417.1), CrY14 (XP_001696992.1), OsY14a (NP_001051661.1), OsY14b (XP_006654975.1), ZmY14a (NP_001150559.1), ZmY14b (NP_001150263.1), SiY14a (XP_004960437.1), SiY14b (XP_004981327.1), BdY14 (XP_003568971.1), SbY14a (XP_002439249.1), SbY14b (XP_002466233.1), RcY14 (XP_002513523.1), PtY14a (XP_002299789.1), PtY14b (XP_002314085.2), CsiY14 (XP_006470660.1), MtY14 (XP_003610955.1), GmY14 (XP_003517521.1), TcaY14 (XP_007015222.1), CsaY14 (XP_004139049.1), StY14a (XP_006353634.1), StY14b (NP_001274809.1), TcrY14 (ABB91897.1), PsY14 (ABK25331.1), PpY14a (XP_001758256.1) and PpY14b (XP_001771298.1)

To obtain information about the evolutionary relationships of HbMAGOs and HbY14s, phylogenetic analyses were done based on multiple sequence alignments of all transcribed proteins from these two gene families in plants. Phylogenetic analysis based on the alignments of MAGO family proteins showed that rubber trees and other dicots were clustered in same group (Figure 2A), whereas members of the Y14 family clustered with other dicots in a single group (Figure 2B). These analyses also revealed that the MAGO and Y14 genes were present in algae and that their differentiation accompanied that of the corresponding species.

### Subcellular localization of HbMAGOs and HbY14s

**Figure 3:**
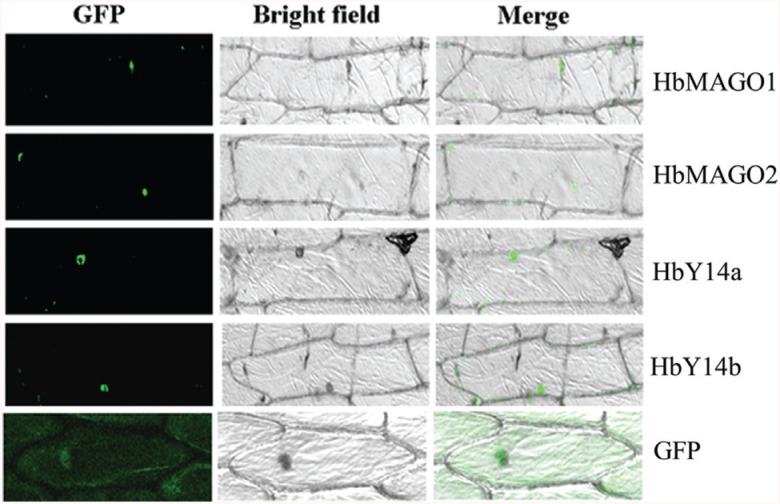
Subcellular localization of HbMAGOs and HbY14s. Bottom row of panels: fluorescence, bright field and merged fluorescence images of the GFP control. The other columns indicate the corresponding fluorescence, bright field and merged fluorescence images of HbMAGOs and HbY14s.

MAGO and Y14 are nucleocytoplasmic shuttling proteins located predominantly in the nucleoplasm and nuclear speckles (Micklem*et al.,* 1997; Kataoka *et al.,* 2000; Mohr *et al.,* 2001). To confirm the subcellular location of HbMAGOs and HbY14s, the fusion proteins and the GFP control constructs were introduced into onion epidermal cells by agroinfiltration using a water circulating vacuum pump and observed under a fluorescence microscope. HbMAGOs and HbY14s were targeted exclusively to the nucleus of the epidermal cells, whereas the GFP control protein was distributed throughout the cells (Figure 3).

### Expression analysis of *HbMAGOs* and *HbY14s*in different tissue*s*

**Figure 4:**
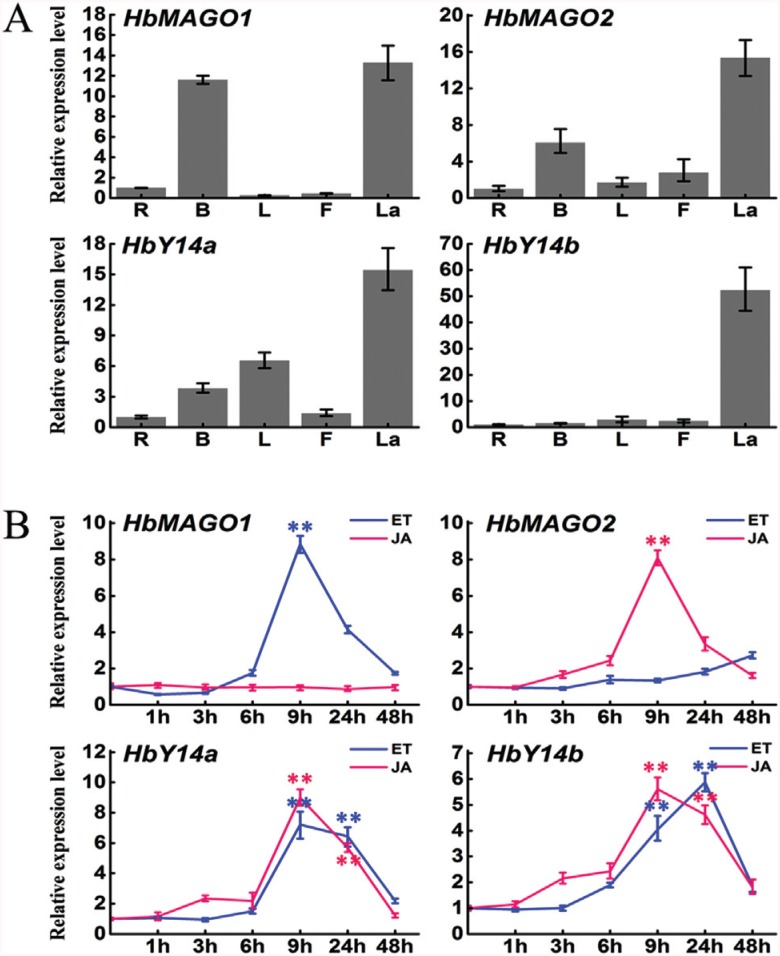
Expression patterns of *HbMAGOs* and*HbY14s* in different tissues (A) and the responses to treatment with jasmonic acid (JA) and ethylene (ET) (B). Relative transcript abundances of *HbMAGOs* and*HbY14s* were examined by RT-qPCR. The Y-axis indicates the relative transcript abundance level, while the X-axis denotes the rubber tree tissues examined (A) and the time course of the response to treatment with ET and JA (B). The rubber tree actin gene (GenBank HQ260674.1) was used as an internal control. PCR primers were designed to avoid the conserved region and to amplify 100-300 bp products. The primer sequences are shown in Table 2. B – bark, L – leaves, F – flowers, La – latex and R – roots. **p < 0.01 (ANOVA).

Real-time quantitative PCR was used to examine the expression patterns of HbMAGOs and HbY14s in bark, flowers, latex, leaves and roots. The expression profiles showed that the four genes (two each for HbMAGO and HbY14) were transcribed in all of the tissues examined, with the highest transcription in latex; for HbMAGO1, the level of transcription in bark was similar to that seen in latex (Figure 4A).

### Expression patterns of *HbMAGOs* and *HbY14s*in latex respond to treatment with JA and ET

Since ET and JA have an important role as signaling molecules that regulate rubber biosynthesis in rubber trees (Hao and Wu, 2000; Zeng *et al.,*2009), we examined whether exposure to ET or JA could influence HbMAGO and HbY14 expression in latex (Figure 4B), despite the lack of evidence relating MAGOs and Y14s to hormone signaling pathways. HbMAGO1 was regulated by ET but not by JA, and HbMAGO2 was induced by JA but not by ET. Two HbY14s were induced by ET and JA in latex. Gene expression increased significantly within 9 h and subsequently decreased when treated with the plant hormones, *i.e.,* HbMAGO1 was influenced by ET, HbMAGO2 by JA, HbY14a by ET and JA, and HbY14b by JA; HbY14b expression also increased significantly within 24 h and subsequently decreased in response to ET.

### Interaction of HbMAGOs with HbY14s

**Figure 5:**
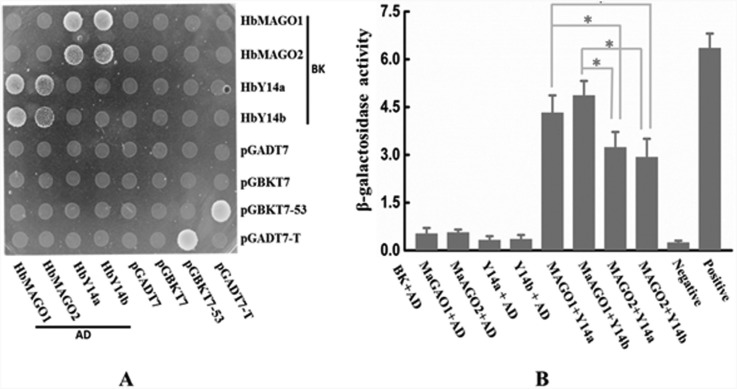
Protein interaction matrices for HbMAGO and HbY14 proteins (A) and quantification of β-galactosidase activity (B). The combination of bait proteins (BD) and prey proteins (AD) is indicated. Interactions between HbMAGOs and HbY14s were detected in yeast and the same amounts of co-transformed yeast cells were grown in the highest stringent conditions (QDO/X/A medium). Transformants containing BK-53 and AD-T were used as positive controls and those containing only BK and AD or fused proteins with BK or AD only were used as negative controls (A). In panel (B), β-galactosidase activity was used as an indicator of the interaction between HbMAGOs and HbY14s (B). Representative results obtained in at least three independent experiments are shown. The columns in panel (B) represent the mean ± SEM. *p < 0.05 (ANOVA).

As the core of EJC, MAGO and Y14 can form hetero-dimers. Figure 5A shows that HbMAGOs interacted with HbY14s in the YTH system. The interaction of HbMAGO1 with HbY14s was significantly greater than that of HbMAGO2 with HbY14s, as assessed by quantifying the β-galactosidase activity (Figure 5B). This finding suggests that HbMAGOl interacted specifically with HbY14s.

### Interaction of HbMAGO2 with gp91^phox^

**Figure 6:**
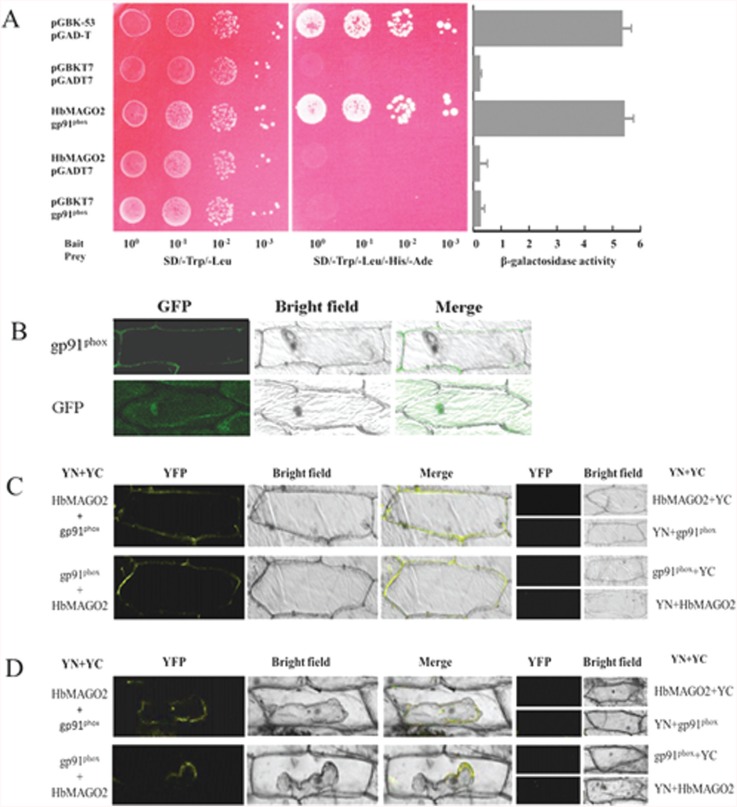
Functional analysis of HbMAGO2. Interaction of HbMAGO2 with gp91^phox^ in yeast (A), subcellular localization of gp91^phox^ (B), and bimolecular fluorescence complementation (BiFC) assays in plants (C,D). pGBK-HbMAGO2 (bait) and pGAD-gp91^phox^ (prey) were co-transformed into AH109 yeast cells. Aliquots (10 ¨L) of a 10 diluted yeast suspension culture co-transformed with bait and prey constructs was spotted onto SD/-Trp/-Leu and SD/-Trp/-Leu/-His/-Ade selection plates. Negative controls consisted of vector with only BK and AD or fused proteins with BK or AD. The intensity of the interaction was assessed by assaying β-galactosidase activity (A). Panel (B) shows the subcellular localization of gp91^phox^. The interaction between HbMAGO2 and gp91^phox^ was confirmed using the BiFC assay (C). Epidermal cells were co-transformed with HbMAGO2 and gp91^phox^proteins fused to the N- or C-terminal half of yellow fluorescent protein. HbMAGO2 or gp91^phox^ with vector were used as negative controls. Onion epidermal cells were observed under normal conditions (C) and after plasmolysis (incubation for 30 min in a 30% sucrose solution) (D). Representative results obtained in at least three independent experiments are shown. The columns in panel (A) represent the mean ± SEM.

Using BK-MAGO as bait, we obtained gp91^phox^ as one of the prey proteins screened from a two-hybrid latex library (Figure 6A). The open reading frame of gp91^phox^ was cloned (GeneBank: AJJZ010935968.1). To confirm the subcellular location of gp91^phox^ proteins, gp91^phox^:GFP fusion proteins were transiently expressed in onion epidermal cells. Confocal imaging of GFP fluorescence revealed that the gp91^phox^:GFP fusion protein was located in the cytomembrane (Figure 6B). The possible interaction between HbMAGO2 and gp91^phox^ was examined using a BiFC assay. A strong fluorescence signal was observed in the cytomembrane of epidermal cells under normal conditions (Figure 6C) and during plasmolysis (Figure 6D), but not with the negative controls. These data confirmed the interaction between HbMAGO2 and gp91^phox^.

## Discussion

There is increasing evidence that many factors involved in basic cellular processes in animals and plants are conserved. MAGO and Y14 proteins, which are important for cellular differentiation in animals, show marked sequence conservation across a wide range of species (Chen *et al.*, 2007). The MAGO and Y14 proteins are encoded by a small gene family in plants. The *Arabidopsis* genome contains one MAGO and one Y14 gene (Park and Muench, 2007), whereas the rice genome contains two MAGO and two Y14 genes (Gong and He, 2014). We have identified two MAGO and two Y14 genes in the rubber tree genome. MAGO and Y14 proteins are highly conserved among eukaryotes, suggesting that the corresponding genes are essential for eukaryotes. Y14 proteins have a well-conserved RBD in the middle region. The RBD is a characteristic feature of the ribonucleo-protein (RNP) family of RNA binding proteins and is evolutionarily conserved (Nagai *et al.*, 1995). The homologs of Y14 that have these characteristics are known to be involved in RNA localization, mRNA splicing and exon-exon junctions (Kim *et al.*, 2001; Mohr *et al.*, 2001; Chuang *et al*., 2013). It has been suggested that HbY14 may also have these critical functions in plants. HbMAGOs and HbY14s shared high amino acid sequence identity with homologs from other species. Although MAGO and Y14 are highly conserved in eukaryotes, the clusters of MAGO and Y14 are easily distinguished between dicots and other clades. The evidence from gene structure also supports this conclusion. The two MAGO genes in humans (MAGOH) consist of five exons with a conserved length and structure, but the coding sequence of MAGOHB is longer than that of MAGOH (Singh *et al.*, 2013).

MAGO and Y14 are nucleocytoplasmic shuttling proteins located predominantly in the nucleoplasm and nuclear speckles (Micklem *et al.*, 1997; Kataoka*et al.*, 2000; Mohr*et al.*, 2001). In *Taiwania cryptomerioides*, TcMAGO-EGFP and TcY14-EGFP were detected at low levels in the cytoplasm at 20 h and 22 h. After 22 h, TcMAGO-EGFP was located only in the nuclei, whereas TcY14-EGFP was present in the nuclei and at low levels in the cytoplasm (Chen *et al.*, 2007). In rice, OsMAGO2 and OsY14b showed identical distributions in cells and were located in the nuclei and cytoplasm. Surprisingly, OsY14a seemed to be uniquely located in the nuclei, in contrast to the established subcellular localization of these EJC sub-units (Gong and He, 2014). As shown here, HbMAGOs and HbY14s were located in the nuclei, in a manner similar to their homologs in other species.

In rubber trees, laticifers are tissues that are specifically involved in the biosynthesis and storage of natural rubber, as well as in defense against pathogens. In this study, we found that HbMAGOs and HbY14s had a similar, constitutive expression pattern in different tissues of rubber tree. Four genes were transcribed in the tissues examined, with the highest transcription occurring in latex, a finding indicative of the vital role of these genes in laticifer cells. HbMAGO1 was regulated by ET but not by JA, and HbMAGO2 was induced by JA but not by ET. In contrast, two HbY14s were regulated by ET and JA in latex. The biosynthesis of natural rubber is enhanced in rubber trees by the endogenous accumulation and exogenous application of JA (Hao and Wu, 2000). Our findings suggest that the HbMAGO and HbY14 genes examined may regulate natural rubber biosynthesis in rubber tree laticifer cells via ET and JA signal transduction pathways. The differences in the levels of transcription of the genes in response to ET and JA also indicated functional differences in laticifer cells. This is the first demonstration that HbMAGO and HbY14 are linked to hormone signaling pathways. The precise relationship will be investigated in future studies.

The MAGO gene was first identified as a strict maternal effect gene in*Drosophila*, where a single point mutant in the MAGO locus gave rise to a grandchildless phenotype because of a defect in the correct cytoplasmic location of oskar mRNA (Boswell *et al.*, 1991; Newmark and Boswell, 1994; Micklem *et al.*, 1997; Newmark*et al.*, 1997). MAGO always functions together with an RNA-binding protein, known as Y14 in *Xenopus* (Kataoka *et al.*, 2000), RBM8A in humans (Zhao *et al.*, 2000) and Tsunagi in *Drosophila* (Mohr *et al.*, 2001); this protein shuttles between the nucleus and cytoplasm (Hachet and Ephrussi, 2001; Kim *et al.*, 2001).

The MAGO-Y14 complex is the core of the EJC assembled on RNA 20 nucleotides upstream of exon-exon junctions (Kataoka *et al.*, 2001; Bono *et al.*, 2004). Determination of the crystal structure of the MAGO-Y14 complex showed that the MAGO-Y14 interaction was highly specific and strongly conserved (Zhao *et al.*, 2000; Kataoka *et al.*, 2001; Le *et al.*, 2001; Shi and Xu, 2003; Stroupe *et al.*, 2006). In plants, the MAGO-Y14 complex has also been found in *T. cryptomerioides* and rice (Chen*et al.*, 2007; Gong and He, 2014). The functional barrier between the two protein families was not observed within dicots or duplicates of rice, but was observed between dicots and monocot or plants and animals (Gong*et al.*, 2014). The high specificity and conservation of the MAGO-Y14 interaction is clade-specific, and such co-evolution allows the interaction between these proteins to be maintained across large evolutionary time scales. In this work, we identified HbMAGO1/2 and HbY14a/b homologs derived from rubber tree latex and confirmed the interaction between HbMAGO and HbY14 using YTH analysis. Overall, our findings indicate that HbMAGO1/2 and HbY14a/b are evolutionarily highly conserved proteins with similar functions to their homologs.

In rubber trees, NADPH (nicotinamide adenine dinucleotide phosphate) oxidase is involved in the accumulation of reactive oxygen species (ROS) in tapping panel dryness (TPD), a physiological disorder characterized by the spontaneous drying up of the tapping cut that results in an abnormally low yield or stoppage of latex flow (Jacob *et al.*, 1994;Chen *et al.*, 2003; Venkatachalam *et al.*, 2009). ROS are involved in the coagulation of rubber particles that dramatically reduces natural rubber production. High ROS production in latex cells triggers oxidative stress leading to the *in situ* coagulation of rubber particles (Chrestin *et al.*, 1984;Li *et al.*, 2010; Leclercq *et al.*, 2012). ROS are also important signaling molecules in plants.

ROS formation is one of the early physiological responses of plant cells to biotic and abiotic stress, such as wounding and pathogens (Bogre *et al.*, 1997; Bolwell *et al.*, 2002). NADPH oxidase is the main source of ROS in plants (Asai *et al.*, 2008). In animals, the NADPH oxidase complex consists of two plasma membrane proteins, gp91^phox^(phox for phagocyte oxidase) and p22^phox^. Cytosolic regulatory proteins p47^phox^, p67^phox^, p40^phox^ and Rac2, translocate to the plasma membrane to form the active complex after stimulation (Brandes *et al.*, 2014). However, no homologs of the p22^phox^, p67^phox^, p47^phox^ and p40^phox^ regulators of phagocyte NADPH oxidase were found in plants.

Plant NADPH oxidases are known as respiratory burst oxidase homologs (RBOHs) and are homologous to the catalytic subunit (gp91^phox^) of mammalian NADPH oxidases (Rodriguez *et al.*, 2007; Suzuki *et al.*, 2011). RBOHs have been identified and characterized in several species, including *Arabidopsis thaliana* (Kawarazaki *et al.*, 2013), potato (Chen *et al.*, 2013), rice (Yoshie *et al.*, 2005), wheat (Yamauchi *et al.*, 2014). In TPD trees, high levels of NADPH oxidase activities lead to the release of O_2_^-^, a toxic form of oxygen (Jacob *et al.*, 1994). Cellular membranes and especially lutoids, which contain coagulant factors involved in the aggregation of rubber particles, are damaged by O_2_^-^ (Chrestin *et al.*, 1984; Li *et al.*, 2010; Leclercq *et al.*, 2012).

In plants, MAGO can participate in biological processes through protein-protein interactions. In *Physalis,* MAGO interacts with a MADS-Domain protein that can regulate plant development through the formation of dimers and higher order complexes to influence male fertility and calyx development (He *et al.*, 2007); this finding suggests that MAGO plays a role in plant biological processes independently of the EJC. Little is known about the function of HbMAGO mediated by interaction with target proteins in rubber trees. As shown here, the YTH and BiFC assays indicated that HbMAGO2 interacted with gp91^phox^. This finding suggests that HbMAGO2 may regulate NADPH oxidase-dependent oxidative bursts in rubber tree latex via protein-protein interactions.

Functional studies have shown that MAGO is involved in development of organization in multicellular organisms. Over-expression or knockdown of MAGO results in phenotypic alterations that vary among plants, including visible longer roots and a more complex root system, a greater number of leaves, short and fasciated stems, short lateral roots and non-viable seeds, and short rice plants with abnormal flowers (Chen *et al.*, 2007;Park *et al.*, 2009; Gong and He, 2014). In*Drosophila*, the EJC controls the splicing of*mapk* and other long intron-containing transcripts and mitogen-activated protein kinase (MAPK) signaling depends on the regulation of MAPK levels by the EJC (Ashton-Beaucage *et al.*, 2010). During development of the eye, MAPK is the primary functional target of MAGO (Roignant and Treisman, 2010). As core components of the EJC linked to MAPK signaling pathway, MAGOs can control animal cell or tissue development. Laticifer cells are some of the most important plant cells that continuously produce natural rubber and are arranged as concentric sheaths in the phloem (Chen *et al.*, 2000; Han*et al.*, 2000; Pickard, 2008). There are few reports on the regulation of laticifer development in general (Tan *et al.*, 2014), and the development of laticifer cells in rubber trees remains poorly understood. Our findings suggest that MAGO may regulate the development of laticifer cells, although further experiments are need to confirm this suggestion.

## Conclusions

The present study has provided a comprehensive genomic analysis of the rubber tree HbMAGO and HbY14 gene families and the first evidence that HbMAGO and HbY14 are linked with hormone signaling pathways, including rubber particle aggregation in laticifer cells. These data provide important insights into the potential roles of HbMAGO and HbY14 gene families in rubber trees and may contribute to clarifying the function of HbMAGO2 in the aggregation of natural rubber particles.
